# Modulation of epithelial sodium channel (ENaC) expression in mouse lung infected with *Pseudomonas aeruginosa*

**DOI:** 10.1186/1465-9921-6-2

**Published:** 2005-01-06

**Authors:** André Dagenais, Diane Gosselin, Claudine Guilbault, Danuta Radzioch, Yves Berthiaume

**Affiliations:** 1 Centre de recherche, Centre hospitalier de l'Université de Montréal/ Hôtel-Dieu, Département de médecine, Université de Montréal, Montreal, Quebec, Canada; 2 Present address: Fonds de solidarité FTQ, Montreal, Quebec, Canada; 3 Departments of Experimental Medicine and Human Genetics, McGill University, Montreal, Quebec, Canada

## Abstract

**Background:**

The intratracheal instillation of *Pseudomonas aeruginosa *entrapped in agar beads in the mouse lung leads to chronic lung infection in susceptible mouse strains. As the infection generates a strong inflammatory response with some lung edema, we tested if it could modulate the expression of genes involved in lung liquid clearance, such as the α, β and γ subunits of the epithelial sodium channel (ENaC) and the catalytic subunit of Na^+^-K^+^-ATPase.

**Methods:**

*Pseudomonas aeruginosa *entrapped in agar beads were instilled in the lung of resistant (BalB/c) and susceptible (DBA/2, C57BL/6 and A/J) mouse strains. The mRNA expression of ENaC and Na^+^-K^+^-ATPase subunits was tested in the lung by Northern blot following a 3 hours to 14 days infection.

**Results:**

The infection of the different mouse strains evoked regulation of α and β ENaC mRNA. Following Pseudomonas instillation, the expression of αENaC mRNA decreased to a median of 43% on days 3 and 7 after infection and was still decreased to a median of 45% 14 days after infection (p < 0.05). The relative expression of βENaC mRNA was transiently increased to a median of 241%, 24 h post-infection before decreasing to a median of 43% and 54% of control on days 3 and 7 post-infection (p < 0.05). No significant modulation of γENaC mRNA was detected although the general pattern of expression of the subunit was similar to α and β subunits. No modulation of α_1_Na^+^-K^+^-ATPase mRNA, the catalytic subunit of the sodium pump, was recorded. The distinctive expression profiles of the three subunits were not different, between the susceptible and resistant mouse strains.

**Conclusions:**

These results show that *Pseudomonas *infection, by modulating ENaC subunit expression, could influence edema formation and clearance in infected lungs.

## Background

The epithelial sodium channel (ENaC) is expressed in epithelial cells of several tissues involved in salt and water reabsorption. The channel is composed of three related subunits (α, β, γ) that are able to reconstitute a functional channel when expressed in *Xenopus laevis *oocytes [1,2]. ENaC is expressed in a wide range of tissues, including the kidney [1,3-5], distal colon [1,3,5], lung [6-8], ear epithelium [9,10], papilla of the tongue [11-13], eyes [14], chondrocytes [15] and differentiating epithelia [16]. ENaC synthesis and activity are highly regulated by hormones, such as aldosterone, vasopressin and catecholamines, by intracellular pH, feedback inhibition and extracellular proteases [17,18]. In the lung, vectorial Na^+ ^transport from the alveoli to the interstitium is the main force that drives water out of the alveoli [19,20]. This transport mechanism plays a crucial role late in gestation and at birth when sodium transport is involved in lung liquid clearance [21]. Its importance at birth has been shown unambiguously in αENaC gene knockout mice, where the inability to clear lung water rapidly leads to hypoxemia and death [22]. Na^+ ^transport is also important in adults for lung liquid clearance [19,23].

Increased ENaC expression has been detected in the lung and in alveolar epithelial cells *in vitro*, following stimulation with steroids, β-agonists, catecholamines, and agents that increase cAMP concentration [24-27]. αENaC expression in the lung is modulated at birth when considerable liquid clearance is required [3,6,27]. It is also upregulated during hyperoxia [28,29] and downregulated during hypoxia, which could explain high altitude lung edema (HALE) [30,31]. Several lines of evidence suggest that up-regulation or downregulation of ENaC activity in the lung could be associated with lung infection. In type I pseudohypoaldosteronism, a recessive genetic disease leading to a non-functional ENaC, susceptibility to lung infection has been reported [32-34]. Although ENaC is not the primary defect associated with cystic fibrosis (CF), airway cells from CF patients show a 2–3-fold increase in Na^+ ^transport compared to normal cells [35,36]. This sodium hyperabsorption results from the inability of cystic fibrosis transmembrane regulator (CFTR) in CF cells to downregulate ENaC activity [37,38].

*Pseudomonas aeruginosa *is a bacterium occuring naturally in a wide range of environments such as in soil, fresh and seawater, plants and decomposing organic matter [39]. Although not usually pathogenic, this common bacterium can evoke opportunistic infections in immunodeficient persons, such as patients with severe burns [39]. *Pseudomonas *can promote nosocomial lung infection after artificial ventilation [40] and is also present in patients with bronchiectasis [39]. Chronic lung infections are the major cause of morbidity and mortality in CF patients [41] where *Pseudomonas aeruginosa *is the main source of chronic lung infection in CF patients [42].

Instillation of *Pseudomonas aeruginosa *in the lung of anaesthetised rabbits has been reported to promote acute pneumonia, resulting in alveolar epithelial injury, loss of epithelial barrier integrity, lung edema, pleural empyema and pleural effusions within 8 h of infection [43]. A more chronic pneumonia model has been developed in the mouse by the intratracheal instillation of *P. aeruginosa *entrapped in agar beads. In this model, the lungs of susceptible mouse strains develop severe lung infection with a strong inflammatory response and some lung edema [44,45]. *Pseudomonas *by itself has been shown to inhibit active sodium absorption in cultured airway epithelial cells [46]. Here, we studied its impact on the expression of genes involved in the modulation of liquid absorption in alveolar and airway epithelium, namely the three ENaC subunits and the catalytic subunit of Na^+^-K^+^-ATPase.

*Pseudomonas *entrapped in agar beads was instilled in the lung of resistant (BalB/c) and susceptible (DBA/2, C57BL/6 and A/J) mouse strains, and the expression of α, β, γENaC and α_1 _Na^+^-K^+^-ATPase mRNA was studied by Northern blotting in lungs infected between 3 hours and 14 days.

## Methods

### Infection of mice with *P. aeruginosa*

Clinical strain 508 of *P. aeruginosa *(provided by Dr. Jacqueline Lagacé, Université de Montréal, Montreal, Canada) was entrapped in agar beads, and 50-μl suspensions containing 2 × 10^5 ^to 1 × 10^6 ^CFU/ml were instilled intratracheally in male mice of resistant (BALB/c) or susceptible (DBA/2, C57BL/6 and A/J) strains as described previously [44,45].

### Macrophage and polymorphonuclear (PMN) counts in bronchoalveolar lavage (BAL)

BAL were performed as described elsewhere with a few modifications [44]. The infected mice were sacrificed by CO_2 _inhalation at different time points after *P. aeruginosa *instillation in the lungs. The trachea was cannulated, and the lungs were washed seven times with 1 ml PBS. Total cell counts were conducted in a hemacytometer. Differential cell counts were made by Diff-Quick staining (American Scientific Products) of Cytospin preparations. Number of animals: day 1, n = 6; day 4, n = 16; day 6, n = 6; day 14, n = 3.

### Northern blotting

The lungs from infected mice were harvested between 3 h to 14 days after infection, homogenized in 5 ml of 4 M guanidine isothiocyanate, and centrifuged on a cesium chloride gradient [44]. Fifteen to 20 μg of total RNA purified from the lungs were electrophoresed on 1% agarose-formaldehyde gel and transferred to Nytran membranes (Schleicher & Schuell, Keene, NH, USA) by overnight blotting with 10 X SSC. Hybridization was performed, as reported previously [3], in Church buffer (0.5 M Na phosphate, pH 7.2, 7% SDS (w/v), 1 mM EDTA, pH 8) [47]. The nylon membranes were hybridized successively with different cDNA probes. (αENaC, βENaC and γENaC, α_1_Na^+^-K^+^-ATPase, glyceraldehyde-3-phosphate dehydrogenase (GADPH) or 18S rRNA). To detect αENaC mRNA, the blots were hybridized with 764-bp mouse αENaC cDNA (His-445 to stop codon) [3]. The probes for rat β and γENaC cDNA were gifts from Dr. B.C. Rossier (Institut de pharmacologie et de toxicologie de l'Université de Lausanne, Lausanne, Switzerland) and coded for the entire cDNA [2]. The α_1_Na^+^-K^+^-ATPase probe was a gift from Dr. J. Orlowski (Physiology Department, McGill University, Montreal, Quebec, Canada) and consisted of a NarI-StuI 332-bp fragment coding from nucleotide 89 to 421 (from the 5'UTR to Arg-61) of the rat kidney and brain α isoform [48]. For quantitative study, αENaC mRNA expression was normalized to murine GADPH with a 455 bp cDNA probe cloned between nucleotide 146 and 601 [44] or with 18S rRNA, using a 640-bp cDNA probe between nucleotidet 852 and 1492 of the rat 18S rRNA sequence [26]. The blots were exposed to Kodak Xar-film with an intensifying screen, or to a PhosphorImager (Molecular Dynamics, Sunnyvale, CA, USA) for densitometric analysis. Because different strains of mice were investigated in this study (BalB/c, DBA/2, C57BL/6 and A/J), the expression of the different mRNA was calculated at each time point as the % of expression relative to an untreated control from the same strain. The data from the different strains were pooled and subjected to statistical analysis.

Between each round of hybridization, the membranes were stripped by treatment with 0.1 X SSC, 1% SDS and 2.5 mM EDTA at 95°C. The blots were allowed to cool gradually with agitation for 30 min at room temperature. The membranes were then rinsed with 5 X SSC and rehybridized. Number of animals: n = between 6 and 8 animals for each time point and each mRNA studied.

### Statistics

For the BAL cell count, the data are presented as means ± SE (standard error). For ENaC and Na^+^, K^+^-ATPase mRNA expresion, the comparisons between groups were analyzed by Wilcoxon signed rank non-parametric test using Statsview software (SAS Institute, Inc., Cary, NC, USA). Probability *p *values < 0.05 were considered to be significant.

## Results

### Inflammation in mice infected with P. aeruginosa

**Figure 1:**
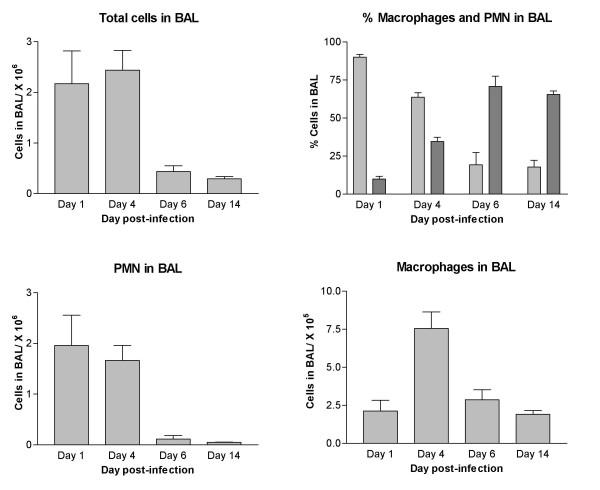
**Differential cell counting in C57BL/6 mice infected with 1–2 × 10^5 ^*Pseudomonas aeruginosa *embedded in agar beads. ***Pseudomonas *infection leads to strong inflammation with recruitment of PMN and macrophages in bronchoalveolar lavage on days 1 and 4 post-infection. Day 1, n = 6; day 4, n = 16; day 6, n = 6; day 14, n = 3. Differential cell counting: PMN in light grey, macrophages in dark grey.

The inflammation process evoked by *Pseudomonas *instillation in the lung of C57BL/6 mice was monitored by studying the number of total cells in BAL at different times after infection. As shown in Figure 1, the inflammation process was more pronounced on days 1 and 4 post-infection. Significant PMN recruitment was noted on day 1 after infection since these cells constituted 90% of the cell population in BAL at that time (Fig. 1). The proportion of PMN decreased gradually over time. On days 6 and 14, there was a significant reduction of PMN in BAL (p < 0.05) compared to day 1. PMN still constituted 18% of the cells in BAL on day 14. The infection also led to modulation in the number of macrophages with a significant increase (p < 0.05) on day 4 post-infection (Fig. 1).

### Modulation of α, β and γENaC expression following lung infection with P. aeruginosa

**Figure 2:**
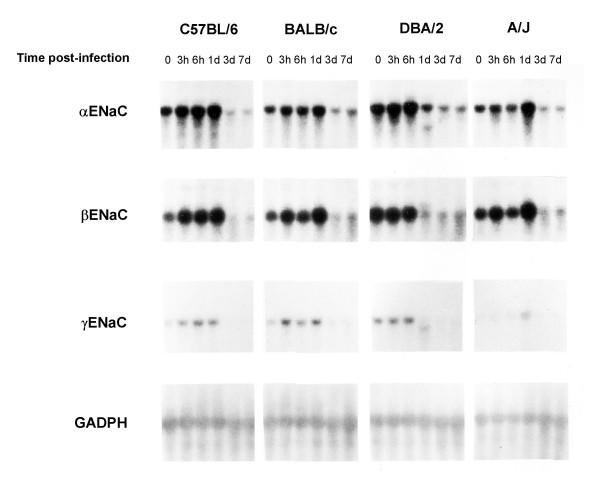
**Expression of α, β and γENaC mRNA in the lung following infection with *Pseudomonas aeruginosa*. **Representative Northern blot of α, β and γENaC mRNA expression following infection with *Pseudomonas *in resistant (BalB/c) and susceptible (DBA/2, C57BL/6 and A/J) strains of mice. There is a characteristic modulation of the three ENaC subunits that is not different between strains.

**Figure 3:**
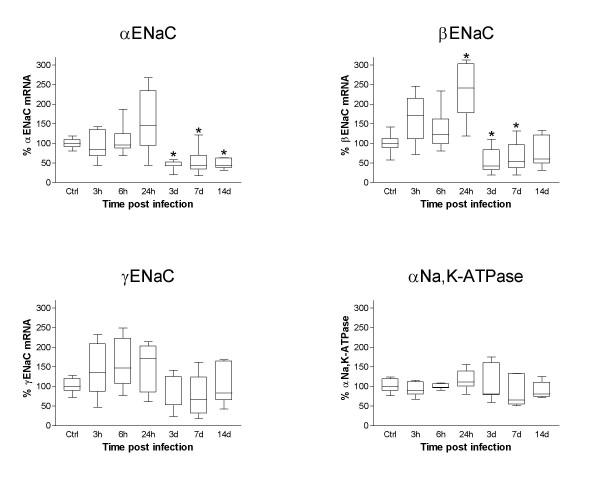
**Densitometric analysis of the modulation of αENaC, βENaC, γENaC and α_1_Na^+^-K^+^-ATPase mRNA following *Pseudomonas *infection. **The modulation of αENaC, βENaC, γENaC and α_1_Na^+^-K^+^-ATPase mRNA by Northern blots hybridization was subjected to a densitometric analysis. Because different strains of mice were investigated in this study (BalB/c, DBA/2, C57BL/6 and A/J), the expression of the different mRNA was calculated at each time point as the % of expression relative to an untreated control coming from the same strain. The α and βENaC mRNA were modulated at some time point by Pseudomonas infection compared to uninfected animals. There was no modulation for γENaC or α_1_Na^+^-K^+^-ATPase mRNA. αENaC mRNA was downregulated compared to uninfected controls on days 3, 7 and 14 post-infection (*, p < 0.05). βENaC mRNA was elevated at 24 h post-infection (*, p < 0.05) compared to uninfected controls and was downregulated thereafter on day 3 and 7 post-infection (*, p < 0.05). Number of animals: n = between 6 and 8 animals for each time point and each mRNA studied.

*Pseudomonas *embedded in agar beads was administered intratracheally in resistant (BALB/c) and susceptible strains of mice (DBA/2, C57BL/6, A/J) as described previously [44,49]. α, β and γENaC expression in infected lungs was measured by Northern blot hybridization (Fig. 2). Expression of the three subunits was highly modulated in time after lung infection, but showed a similar pattern between the four mouse strains tested. The BALB/c strain that is resistant to *Pseudomonas *infection [45], as well as the DBA/2, C57BL/6 and A/J susceptible strains, showed increased α, β and γENaC expression at 24 h, followed by a decrease on day 3 post-infection. The GADPH standard gene did not manifest any modulation of its expression. Densitometric quantitative analyses of the Northern blots were performed for the four mouse strains. The relative expression at each time point was determined relative to uninfected animals of the same strain and the data from the 4 strains were pooled for analysis (Fig. 3). αENaC mRNA expression presented a significant decline to a median of 43% on days 3 and 7 post-infection, and was still decreased to a median of 45% on day 14 post-infection compared to uninfected controls (p < 0.05, Fig. 3). βENaC mRNA expression was increased to a median of 241% of uninfected control values, 24 h post-infection (p < 0.05), and was followed by a decrease to medians of 42% and 54% on day 3 and 7 post-infection (p < 0.05) (Fig. 3). Although the expression of γ ENaC mRNA showed an expression pattern very similar to the α and βENaC subunits, with an increased expression at 24 h (median of 171%) followed by a decreased expression on day 3 (median of 53%) and 7 (median of 66%) of infection, these changes failed however to reach significance (Fig. 3). No modulation of α, β or γENaC mRNA was detected when the lungs were instilled with agarose beads only (data not shown). We also investigated the expression of α_1 _Na^+^-K^+^-ATPase mRNA coding for the catalytic domain of the sodium pump, but could not find any significant change during infection (Fig. 3).

## Discussion

The instillation of *Pseudomonas *enmeshed in agarose beads in the lung is a good model to study lung inflammation [44,45] and lung injury [43] secondary to an infection. For this study, *P. aeruginosa *enmeshed in agarose beads was instilled into the mouse lung because the model allows the development of chronic lung infection in susceptible mouse strains [44,45]. The infection leads to cellular infiltration and alveolar edema that stand on day 3 post-infection and that can be still demonstrated on day 14 post-infection in *Pseudomonas*-susceptible mouse strains [45]. Because the lung inflammation associated with *Pseudomonas *infection is accompanied by lung injury [43], and because we have shown recently that ENaC expression can be modulated under conditions that promote lung injury [50], we tested here if *Pseudomonas *was affecting the mRNA expression level of the three ENaC subunits as well as the catalytic subunit of the Na^+ ^pump since these elements are involved in lung liquid balance across the alveolar epithelium [19,23]. The results reported here indicate that *Pseudomonas *infection modulated the expression of the three ENaC mRNA with a characteristic pattern. There was no significant difference, however, in the expression profile of ENaC mRNA between the *Pseudomonas*-resistant (Balb/C) and -susceptible (DBA/2, C57BL/6, A/J) mouse strains. Modulation of ENaC expression is therefore most likely not a genetic marker linked to the susceptibility of mouse strains to establishment of a chronic infection with *Pseudomonas*.

*Pseudomonas *infection affected ENaC mRNA with a pattern consisting of increased expression at 24 h, followed by a marked decrease on day 3 post-infection. The change in ENaC mRNA was related to bacterial infection, since agarose beads alone failed to evoke any modulation of these RNA. The three ENaC subunits were modulated with a similar profile, with some noticeable differences, however. Although αENaC mRNA expression tends to increase by day 1, the most noticeable feature brought by *Pseudomonas *infection to αENaC mRNA was the significant decreases after 3 days, 7 days and 14 days post-infection. To the best of our knowledge, this is the first report demonstrating that bacterial infection *in vivo *can lead to modulation of ENaC mRNA expression. Recently, αENaC mRNA expression was found to be downregulated in the mouse lung after 7 and 14 days of adenoviral infection [51]. Furthermore, there is some evidence that αENaC expression is also decreased in other models of lung injury. Folkesson et al. [52] reported a decline in ENaC expression following subacute lung injury, 10 days after intratracheal administration of bleomycin. More recently, we recorded a decrease in ENaC expression after ischemia-reperfusion lung injury [50]. All these results, and the results reported in the present report, suggest that the modulation of ENaC expression associated with lung infection could be a widespread mechanism, not specific to a given pathogen or injury process, but a general response of the lung to inflammation and injury.

The β ENaC subunit was also modulated by *Pseudomonas *infection. There was a significant increase in the mRNA expression on day 1 post-infection, followed, as for αENaC, by a decreased expression on day 3 and day 7 post-infection. Different stoichiometries have been proposed for ENaC. One model suggests a 2α, β, γ ratio [53,54] whereas others postulate an octomeric [55] or nonameric structure [55,56]. Although the expression of the α subunit alone is sufficient to allow ENaC activity [1], the three subunits are needed to get a fully functional channel [2]. The expression of the three subunits in *Xenopus laevis *oocytes increases amiloride-sensitive Na^+ ^current by 100% compared to αENaC alone [2]. The α, β and α, γ channels are 20 times less effective in driving amiloride-sensitive current than the native channels and show differences in their biophysical properties [57,58]. Gene inactivation or over-expression of the different ENaC subunits has revealed important differences in the role each subunit plays in lung liquid management. αENaC knockout mice develop respiratory distress and die within 40 h from birth because of their inability to clear lung liquid [22]. Lung liquid clearance at birth is also slower in γENaC knockout mice [59], but is not affected in βENaC knockouts [60]. Increased transgenic expression of βENaC targeted in the airway epithelia, but not α or γ subunits, showed an increase Na^+ ^transport across the airway epithelium and a reduced height of the airway surface liquid [61]. For all these reasons, it is difficult to predict how the modulation of ENaC mRNA expression and its effect on the ratio of the three subunits, would have an impact on ENaC activity. In addition, ENaC mRNA content also does not necessarily reflect the amount of active channel at the membrane. One thing seems clear however, because of its prominence in the lung, the diminution of αENaC expression that we detected in the lung following *Pseudomonas *infection, could certainly influence amiloride-sensitive current and lung liquid clearance as in αENaC KO mice rescued by transgenic expression of αENaC that has a lower expression of ENaC in the lung [62,63]. In such model, there is a reduced ENaC current in tracheal cells [63], and a much slower lung liquid clearance following thiourea or hyperoxia-induced lung edema [64].

The general biphasic modulation of ENaC mRNA expression with an increase at 24 h followed by a decrease thereafter is an interesting finding that could explain some contradictory reports concerning ENaC expression in lung following *Pseudomonas *infection. Acute bacterial pneumonia in rats has been shown to increase alveolar epithelial fluid clearance [65,66] when in late pneumonia, there is a decrease in the lung liquid clearance ability of the lung [66]. These contradictory results could be well explained by the modulation of ENaC expression reported here. The long term ENaC downregulation by *Pseudomonas *infection could be of potential clinical significance to understand the slow improvement in some ARDS patients.

In contrast to α and β ENaC, α_1 _Na^+^-K^+^-ATPase mRNA was unaffected in the course of lung infection. This is similar to what has been reported during adenovirus lung infection [51] where αENaC, aquaporin 1 (AQP1) and AQP5 mRNA show decreased expression, but not α_1 _Na^+^-K^+^-ATPase. In ischemia reperfusion injury, there was also no modulation of α_1 _Na^+^-K^+^-ATPase expression despite significant ENaC downregulation [50]. These results, as well as the data reported here, suggest that the inflammatory process seems to selectively affect, and not in a non-specific way, some elements of lung liquid clearance. It would be difficult at this time to speculate on the reasons for this modulation. Na^+^-K^+^-ATPase is an important element in lung liquid clearance, however, by being one of the key generator of membrane potential, the enzyme also affects other channels and ion transport process. It is possible that by modulating ENaC expression and not α_1 _Na^+^-K^+^-ATPase, *Pseudomonas *infection alters the Na^+ ^transport system but does not change other important cell functions meditated by Na^+^-K^+^-ATPase. Furthermore, despite a similar mRNA expression level, there could be a fall in protein content or activity of the sodium pump. Additional experiments are necessary to answer this question.

Several studies report that in lung epithelial cells, viral infection [67,68], mycoplasma [69], bacterial infection [70,71], and inflammatory cytokines such as tumor necrosis factor-α (TNF-α) [70,72], interleudin-1β (IL-1β) [73], or TGF-β [74] decrease the expression of water channels, such as AQP1 and AQP5 and reduce the short circuit current generated by cells. Adenoviral lung infection in mice results in pulmonary inflammation and lung edema with lowered expression of AQP1, AQP5 and αENaC [51]. All these data, including the results presented here, suggest that lung inflammation, by decreasing the expression of αENaC and water channels, could hamper the liquid clearance ability of the lungs and favour edema formation.

## Conclusions

We have shown in this report that *Pseudomonas *infection modulates ENaC mRNA expression. This modulation is independent of mouse strain susceptibility to establishment of chronic infection with *Pseudomonas*. Although there is an elevation of ENaC expression after 24 h, the most important feature is probably the long-lasting decrease of αENaC transcripts on days 3 and 7 post-infection. The lung inflammation induced by *Pseudomonas *infection therefore seems to favour a reduction in the expression of an essential element involved in lung liquid clearance as well as the regulation of airway surface liquid volume.

## Authors' contributions

AD performed the hybridization, the statistical analysis of the blots and wrote the manuscript. DG performed the *Pseudomonas *instillation, RNA extraction and Northern blotting of RNA sample. The BAL recovery as well as PMN and macrophage counting was performed by CG. YB and DR designed and co-ordinated the study. All authors read and approved the final manuscript.
